# PL-hMSC and CH-hMSC derived soluble factors inhibit proliferation but improve hGBM cell migration by activating TGF-β and inhibiting Wnt signaling

**DOI:** 10.1042/BSR20231964

**Published:** 2024-05-17

**Authors:** Tanawat Uthanaphun, Sirikul Manochantr, Chairat Tantrawatpan, Duangrat Tantikanlayaporn, Pakpoom Kheolamai

**Affiliations:** 1 Master of Science Program in Stem Cell and Molecular Biology, Faculty of Medicine, Thammasat University, Pathumthani 12120, Thailand; 2 Center of Excellence in Stem Cell Research and Innovation, Faculty of Medicine, Thammasat University, Pathumthani 12120, Thailand; 3 Division of Cell Biology, Faculty of Medicine, Thammasat University, Pathumthani 12120, Thailand

**Keywords:** cell migration, cell proliferation, glioma, mesenchymal stem cell, paracrine signalling, placenta

## Abstract

Glioblastoma multiforme (GBM) is one of the most common and aggressive brain tumors. GBM resists most chemotherapeutic agents, resulting in a high mortality rate in patients. Human mesenchymal stem cells (hMSCs), which are parts of the cancer stroma, have been shown to be involved in the development and progression of GBM. However, different sources of hMSCs might affect GBM cells differently. In the present study, we established hMSCs from placenta (PL-hMSC) and chorion (CH-hMSC) to study the effects of their released soluble factors on the proliferation, migration, invasion, gene expression, and survival of human GBM cells, U251. We found that the soluble factors derived from CH-hMSCs and PL-hMSCs suppressed the proliferation of U251 cells in a dose-dependent manner. In contrast, soluble factors derived from both hMSC sources increased U251 migration without affecting their invasive property. The soluble factors derived from these hMSCs decreased the expression levels of *CyclinD1, E2Fs* and *MYC* genes that promote GBM cell proliferation but increased the expression level of *TWIST* gene, which promotes EMT and GBM cell migration. The functional study suggests that both hMSCs might exert their effects, at least in part, by activating TGF-β and suppressing Wnt/β-catenin signaling in U251 cells. Our study provides a better understanding of the interaction between GBM cells and gestational tissue-derived hMSCs. This knowledge might be used to develop safer and more effective stem cell therapy that improves the survival and quality of life of patients with GBM by manipulating the interaction between hMSCs and GBM cells.

## Background

Glioblastoma multiforme (GBM) is the most common and aggressive brain tumor, accounting for 45.2% of all primary malignant brain tumors and 54% of all gliomas [1,2]. GBM can occur *de novo* as a primary GBM or develops from a pre-existing astrocytoma to become a secondary GBM. Despite several therapeutic modalities, such as surgery and chemoradiation therapy, most patients with GBM experience relapse within one year, resulting in a low survival rate [3,4]. GBM forms a solid tumor consisting of GBM cells and several cancer stromal cells, including endothelial cells, cancer-associated fibroblasts (CAFs), and various immune cells [5,6]. Complex interactions between GBM cells, cancer stromal cells, and the surrounding extracellular matrix create a tumor microenvironment (TME) that facilitates growth, increases angiogenesis and promotes invasion of GBM cells [7–9]. Furthermore, TME also contributes to GBM resistance to chemotherapeutic agents and immunotherapy by modulating the patient’s immune response and reducing drug penetration into the tumor [7,10].

Therefore, the complex interactions between GBM cells and their associated cancer stromal cells must be unraveled to develop more effective treatments. One of the main cancer stromal cells in the GBM is mesenchymal stromal cells (MSCs). MSCs are multipotent stem/progenitor cells that have been isolated from various tissues [11,12]. Due to their ability to graft into a tumor (a property called ‘tumor tropism’) and release several bioactive molecules, human MSCs (hMSCs) have been exploited for many clinical applications, including cancer treatment. Previous studies showed that hMSCs could migrate through the blood–brain barrier and become part of the GBM stroma, either by further differentiating to CAFs or becoming GBM-associated MSCs (GA-MSCs) that play an essential role in the regulation of GBM properties [11,13]. However, the effects of hMSCs on the properties of GBM remain controversial. Some studies showed that hMSCs inhibited GBM growth and invasion [14–16], while others reported that hMSCs enhanced GBM cell proliferation and invasion [17–20]. These conflicting results are likely due to the various sources of hMSCs and cancer cells used in each study, which makes the results of hMSC therapy in cancer patients unreliable and difficult to predict.

While most studies use hMSCs derived from bone marrow (BM-hMSCs) and adipose tissue (Ad-hMSCs) in their research and clinical applications, the derivation of hMSCs from these tissues requires an invasive procedure and the number of isolated hMSCs is usually limited. Therefore, in the present study, we established hMSCs from chorion and placental tissues (CH-hMSCs and PL-hMSC, respectively), which are easily obtained in large numbers using a non-invasive procedure. Furthermore, several previous studies, including ours, have shown that CH-hMSCs and PL-hMSCs have a great proliferative capacity and release several beneficial soluble factors that suppress proliferation of many types of cancer cells [21–25]. CH-hMSCs and PL-hMSCs can also be collected from a large number of donors with diverse genetic backgrounds to test their effect on various diseases and find the appropriate hMSC donor for each patient. Although CH-hMSCs and PL-hMSCs exhibit typical characteristics of hMSCs similar to those of BM-hMSCs, their effects on GBM cell properties have not yet been investigated. Due to the heterogeneity of hMSCs, hMSCs derived from different tissues or donors are likely to release different factors that affect the properties of GBM cells differently. Therefore, in the present study, our objective was to investigate the effects of CH-hMSCs and PL-hMSCs derived from at least three donors on the proliferative, survival, migratory, and invasive capacities of human GBM cells, as well as the signaling pathways mediating these effects. Understanding mechanisms that mediate the interaction between GBM cells and hMSCs will advance our knowledge regarding the roles of hMSCs in the growth and progression of GBM. This knowledge could then be used to develop more effective stem cell therapy for patients with GBM by modulating the interaction between hMSCs and GBM cells.

## Methods

### Subjects

The present study was approved by the Ethics Committee for Human Research of the Faculty of Medicine, Thammasat University (project number: MTU-EC-DS-1-055/64). The study was carried out according to the World Medical Association Declaration of Helsinki and ICH-GCP. Healthy mothers donated their placentas and chorion after giving birth. All subjects gave their informed consent.

### Isolation and culture of hMSCs

As described in our previous study [23], placenta and chorion were separated from each other, cut into small pieces, and incubated for 30 min at 37°C in 0.25 percent (w/v) trypsin-EDTA (Thermo Fisher Scientific, U.S.A.). After being washed twice with PBS, digested tissues were resuspended in DMEM + 10% (v/v) Fetal Bovine Serum (FBS) (Thermo Fisher Scientific, U.S.A.) and cultured in 25 cm^2^ flasks (Corning, U.S.A.) at 37°C in a humidified environment containing 5% CO_2_. Every three days, the medium was replaced and the cells were subcultured when their confluence reached 80% to facilitate further expansion. The hMSCs were examined using a phase-contrast microscope (Nikon Eclipse Ts2R, Japan).

### Culture of human glioblastoma cells

The human glioblastoma cell line U251 was purchased from the European Collection of Authenticated Cell Cultures (ECACC, U.K.). Cells were expanded in DMEM (Thermo Fisher Scientific, U.S.A.) supplemented with 10% (v/v) FBS (Thermo Fisher Scientific, U.S.A.) and 1% (v/v) GlutaMAX™ (Thermo Fisher Scientific, U.S.A.) at 37°C in a humidified atmosphere containing 5% CO_2_ and the medium was replaced every 2 days during the culture period. When the cell density reached approximately 80% confluence, they were passaged to facilitate further expansion.

### Immunophenotyping of hMSCs by flow cytometry

As described in our previous study [23], approximately 4 × 10^5^ hMSCs (passage 3–5th) were resuspended in 50 μl PBS and treated with 10 μl of the following fluorochrome-labeled mouse anti-human monoclonal antibodies for 30 min at 4°C in the dark: anti-CD45-FITC (BD Pharmingen, U.S.A.), anti-CD34-PE (Biolegend, U.S.A.), anti-CD90-FITC (AbD Serotec, U.S.A.), and anti-CD73-PE (Biolegend, U.S.A.). Cells were then washed twice with PBS before being fixed with 1% (w/v) paraformaldehyde in PBS (Sigma-Aldrich, U.S.A.). The flow cytometry was carried out using CellQuest™ software and a FACScalibur™ flow cytometer (Becton Dickinson, U.S.A.).

### Osteogenic and adipogenic differentiation of hMSCs

As described in our previous study [23], hMSCs (passage 3–5th) were seeded in a 6-well plate (Corning, U.S.A.) at a density of 4.5 × 10^3^ cells/cm^2^ and cultured in DMEM + 10% (v/v) FBS until their confluence reached 80%. For osteogenic differentiation, the medium was replaced with osteogenic differentiation medium [DMEM supplemented with 100 nM dexamethasone (Sigma-Aldrich, U.S.A.), 10 mM β-glycerophosphate (Sigma-Aldrich, U.S.A.), and 50 µg/ml ascorbic acid (Sigma-Aldrich, U.S.A.)]. Cells were cultured for 28 days with a media replacement every 3 days. On day 28, the cells were washed twice with PBS, fixed with 10% (v/v) formaldehyde for 15 min at room temperature, washed twice with distilled water, and stained with 40 mM Alizarin Red S (Sigma-Aldrich, U.S.A.) for 20 min at room temperature. For adipogenic differentiation, cells were cultured in adipogenic differentiation medium [DMEM supplemented with 2.5 mM glucose (Sigma-Aldrich, U.S.A.), 0.5 mM isobutyl methylxanthine (IBMX; Sigma-Aldrich, U.S.A.), 1 µM dexamethasone (Sigma-Aldrich, U.S.A.), 10 µM insulin (Sigma-Aldrich, U.S.A.) and 0.2 mM indomethacin (Sigma-Aldrich, U.S.A.)], for 28 days with media replacement every 3 days. On day 28, the cells were washed twice with PBS, fixed with vaporized 37% formalin for 10 min at room temperature, washed twice with distilled water, and stained with 0.5% (w/v) Oil Red O (Sigma-Aldrich, U.S.A.) in 6% (v/v) isopropanol for 20 min at room temperature. After staining, cells were examined with a phase contrast microscope (Nikon Eclipse Ts2R-FL, Japan).

### Preparation of hMSC-conditioned medium (hMSC-CM)

As described in our previous study [21], hMSCs were cultured in 175 cm^2^ culture flasks (SPL Life Sciences, Korea) with DMEM + 10% (v/v) FBS until their density reached 90% confluence. At this stage, the medium was replaced and the cells were incubated with 15 ml of serum-free DMEM for 24 h. After incubation, the conditioned medium (CM) was collected, concentrated using an Amicon® Ultra-15 centrifugal filter unit with a molecular weight cut-off of 10 kDa (Millipore, U.S.A.), filtered through a 0.22 µm filter (Millipore, U.S.A.) and stored at −80°C for later use.

### *In vitro* proliferation assay

As described in our previous study [23], to evaluate the effect of hMSC-derived soluble factors on GBM cell proliferation, U251 cells were seeded in a 96-well plate [SPL Life Science, Korea] at a density of 1 × 10^3^ cells/well in DMEM + 10% (v/v) FBS and cultured for 12 h. At this stage, the cells were separated into three groups. In the first group (CH-CM), the medium was replaced with 10%, 25%, 50% or 75% (v/v) CH-hMSC-CMs in DMEM + 10% (v/v) FBS. In the second group (PL-CM), the medium was replaced with 10%, 25%, 50%, or 75% (v/v) PL-hMSC-CMs in DMEM + 10% (v/v) FBS. In the third group that serves as the control, the medium was replaced with 10%, 25%, 50%, or 75% (v/v) U251-CM in DMEM + 10% (v/v) FBS. Cells in each group were further cultured for 5 days and the number of cells was determined using the 3-(4,5-dimethylthiazol-2-yl)-2,5-diphenyltetrazolium bromide (MTT) assay (Sigma-Aldrich, U.S.A.), according to the manufacturer’s instructions at an interval of 24 h. At the measurement time, the medium was discarded, 100 µl of 2 mg/ml MTT solution was added to each well, and the plate was incubated at 37°C for 3 h. After incubation, the solution was removed and 100 µl dimethyl sulfoxide (DMSO; VWR Chemicals, France) was added to dissolve the magenta crystal of Formazan. Once the crystal is completely dissolved, the absorbance of each well is measured at 570 nm using a Microplate Reader (BioTex, U.S.A.).

### *In vitro* migration and invasion assays

As described in our previous study [23], to investigate the effect of soluble factors derived from hMSCs on the migration of GBM cells, U251 cells were co-cultured with CH-hMSCs and PL-hMSCs using a transwell culture system to allow an indirect interaction between U251 cells and hMSCs through the soluble factors released from both populations. The hMSCs were seeded in a 24-well plate [SPL Life science, Korea] at a density of 5 × 10^4^ cells/well in 500 µl DMEM + 10% (v/v) FBS and cultured for 12 h. At this stage, 5 × 10^4^ serum-starved U251 cells (previously incubated in serum-free DMEM for 12 h) resuspended in 250 µl complete growth medium were seeded into a transwell insert with a 8 µm pore polycarbonate membrane (Corning, U.S.A.). The transwell inserts were then placed in the 24-well plates containing hMSCs, the volume of growth medium in the lower chamber was adjusted to 750 µl. After 24 h of culture, the transwell inserts were removed and fixed with 2% (v/v) paraformaldehyde in PBS. After fixing, non-migratory cells that remained on the upper side of the transwell membrane were removed by a cotton bud. The membranes were then stained with 0.5% (w/v) Hoechst 33342 in PBS (Sigma-Aldrich, U.S.A.) and examined with a fluorescence microscope (Nikon Eclipse Ts2R-FL, Nikon, Japan). For the invasion assay, the procedure was the same as that used for the migration assay, except that the transwell inserts were pre-coated by incubation with 100 µl of 0.3 mg/ml Matrigel (Corning, U.S.A.) at 37°C for 18 h before U251 cell seeding.

### Apoptosis assay

To evaluate the effect of soluble factors derived from hMSCs on the survival of GBM cells, U251 cells were co-cultured with CH-hMSCs and PL-hMSCs using a transwell. U251 cells were seeded in a 24-well plate at a density of 3 × 10^4^ cells/well in 500 µl DMEM + 10% (v/v) FBS and cultured for 12 h. At this stage, 3 × 10^4^ hMSCs resuspended in 250 µl DMEM + 10% FBS were seeded into a transwell insert with an 8 µm pore polycarbonate membrane (Corning, U.S.A.). The transwell inserts were then placed in the 24-well plates containing U251 cells, the volume of growth medium in the lower chamber was adjusted to 750 µl. After 72 h of culture, transwell inserts were discarded, U251 cells were harvested, and percentages of apoptotic U251 cells were determined using the APC Annexin V Apoptosis Detection Kit with PI (Biolegend, U.S.A.) according to the manufacturer’s instructions. Signal detection was performed by FACS Calibur™ (Becton Dickinson, U.S.A.) using Cell Quest® software (Becton Dickinson, U.S.A.).

### Quantitative real-time PCR (qRT-PCR)

As described in our previous study [23], a gene expression study was performed using qRT-PCR. A complete list of primers is provided in Table 1. U251 cells were seeded in a 6-well plate at a density of 1 × 10^5^ cells/well and cultured in DMEM + 10% (v/v) FBS until their confluence reached 90%. Once the target confluence was reached, the medium was replaced with fresh 75% (v/v) hMSC-CMs in DMEM + 10% (v/v) FBS and the cells were cultured for another 72 h. U251 cells cultured in 75% (v/v) U251-CMs in DMEM + 10% (v/v) FBS served as controls. At the end of the culture, total RNA was isolated from treated U251 cells using TRIzol™ reagent (Sigma-Aldrich, U.S.A.), according to the manufacturer’s instructions. The isolated RNAs were then used to determine the expression levels of target genes by iTaq Universal One-Step RT-qPCR Kits (Bio-rad, U.S.A.), according to the manufacturer’s instruction. PCR was carried out using an Applied Biosystems 7500 Fast Real-Time PCR system (Applied Biosystem, U.S.A.) with the following setting: initial denaturation at 95°C for 10 min, followed by 40 cycles of denaturation (95°C, 10 s), annealing (60°C, 10 s), and extension (72°C, 40 s). The mRNA level of each target gene was normalized with the mRNA level of glyceraldehyde-3-phosphate dehydrogenase (*GAPDH*) using a 7500 software version 2.0.5 (Applied Biosystem, U.S.A.).

**Table 1:** List of primers

Genes of interest	Sequences	
	Forward primer (5′→3′)	Reverse primer (5′→3′)
*NOTCH1*	TCACGCTGACGGAGTACAAG	CCACACTCGTTGACATCCTG
*NOTCH2*	TCAGCCGGGATACCTATGAG	GTAGGAACCAGGCAGGTTGA
*NFKB1*	CTGGAAGCACGAATGACAGA	TGAGGTCCATCTCCTTGGTC
*NFKB2*	TGCACTGCTTCAGAGTGGAG	GGCTAGATGCAAGGCTGTTC
*PROM1*	GGGTCTTGAATTCCATTGGTT	AAATCACGATGAGGGTCAGC
*ITGA1*	GCCGGATTGTTGCTGTTAAT	ATTTGAGGCAAACCTGAGGA
*E2F1*	GCCAAGAAGTCCAAGAACCA	CAGTGTCCTCGGAGAGCAG
*E2F2*	GGCCAAGAACAACATCCAGT	CGTGTTCATCAGCTCCTTCA
*MYC*	TTTCGGGTAGTGGAAAACCA	CAGCAGCTCGAATTTCTTCC
*CyclinD1*	CGTGGCCTCTAAGATGAAGG	CTGGCATTTTGGAGAGGAAG
*TWIST*	GGCTCAGCTACGCCTTCTC	CACGCCCTGTTTCTTTGAAT
*GAPDH*	GTCAACGGATTTGGTCGTATTG	CATGGGTGGAATCATATTGGAA

### Study the roles of TGF-β, Wnt/β-catenin, and NF-kB signaling pathways in the effects of hMSC-derived soluble factors

To elucidate the molecular mechanisms underlying the effects of soluble factors derived from hMSCs on the GBM cell properties, specific small molecules that modified the transforming growth factor-beta (TGF-β), Wnt/β-catenin, and nuclear factor kappa-light-chain-enhancer of activated B cells (NF-kB) signaling pathways were added to the hMSC/U251 co-culture to investigate the roles of these pathways in the interaction between these two cell types. To study the roles of the TGF-β, Wnt/β-catenin, and NF-kB signaling pathways in the suppressive effects of hMSC-derived soluble factors on GBM cell proliferation, 1 × 10^3^ U251 cells were seeded in a 96-well plate in DMEM + 10% (v/v) FBS and cultured for 12 h. At this stage, the cells were separated into four groups. In the first group (CH + CHIR and PL + CHIR), the medium was replaced with 75% (v/v) hMSC-CMs supplemented with 5 µM ChiR99021, a Wnt activator (Sigma-Aldrich, U.S.A.). In the second group (CH + SB and PL + SB), the medium was replaced with 75% (v/v) hMSC-CMs supplemented with 2 µM SB431542, a TGF-β inhibitor (Sigma-Aldrich, U.S.A.). In the third group (CH + BA and PL + BA), the medium was replaced with 75% (v/v) hMSC-CMs supplemented with 7 µM betulinic acid (BA), an NF-kB activator (Sigma-Aldrich, U.S.A.). In the fourth group, which serves as control, the medium was replaced with 75% (v/v) hMSC-CMs without small molecule supplementation. The cells in each group were further cultured for 5 days and the number of cells was determined by an MTT assay.

To study the roles of TGF-β, NF-kB, and Wnt/β-catenin signaling pathways in the effects of soluble factors derived from hMSC on GBM cell migration, U251 cells were co-cultured with CH-hMSCs and PL-hMSCs using a transwell culture system in the presence of 5 µM ChiR99021, 2 µM SB431542, or 7 µM betulinic acid for 24 h. The small molecules are used individually and in combination as a treatment condition. Cell migration was then determined by the migration assay. U251 cells co-cultured with hMSCs without small molecule supplementation served as controls.

To study the roles of TGF-β, Wnt and NF-kB signaling pathways in the effects of soluble factors derived from hMSC on viability and apoptosis of GBM cells, U251 cells were co-cultured with CH-hMSCs and PL-hMSCs using a transwell culture system in the presence of 5 µM ChiR99021, 2 µM SB431542, or 7 µM betulinic acid for 72 h. Small molecules are used both individually and in combination as a treatment condition. U251 cells co-cultured with hMSCs without small molecule supplementation served as controls. The percentages of viable and apoptotic cells were determined by the apoptosis assay.

### Statistical analysis

Data were presented using the mean and standard error of the mean (SEM). The unpaired Student’s *T*-test was used to assess the significance of the variations in the observed data. A *P*-value <0.05 was used to indicate statistical significance.

## Results

### Characteristics of chorion- and placenta-derived hMSCs

In accordance with the minimal standards recommended by the International Society for Cell Therapy (ISCT) [26], CH-hMSCs and PL-hMSCs established in the present study showed typical hMSC characteristics. They had fibroblast-like morphology (Supplementary Figure S1A), exhibited common hMSC surface markers (CD73, CD90, and CD105) and did not express hematopoietic surface markers, CD34 and CD45 (Supplementary Figure S1B), and were capable of differentiation into adipocytes and osteoblasts (Supplementary Figure 1C and 1D).

### The effect of soluble factors derived from hMSCs on U251 cell proliferation

**Figure 1:**
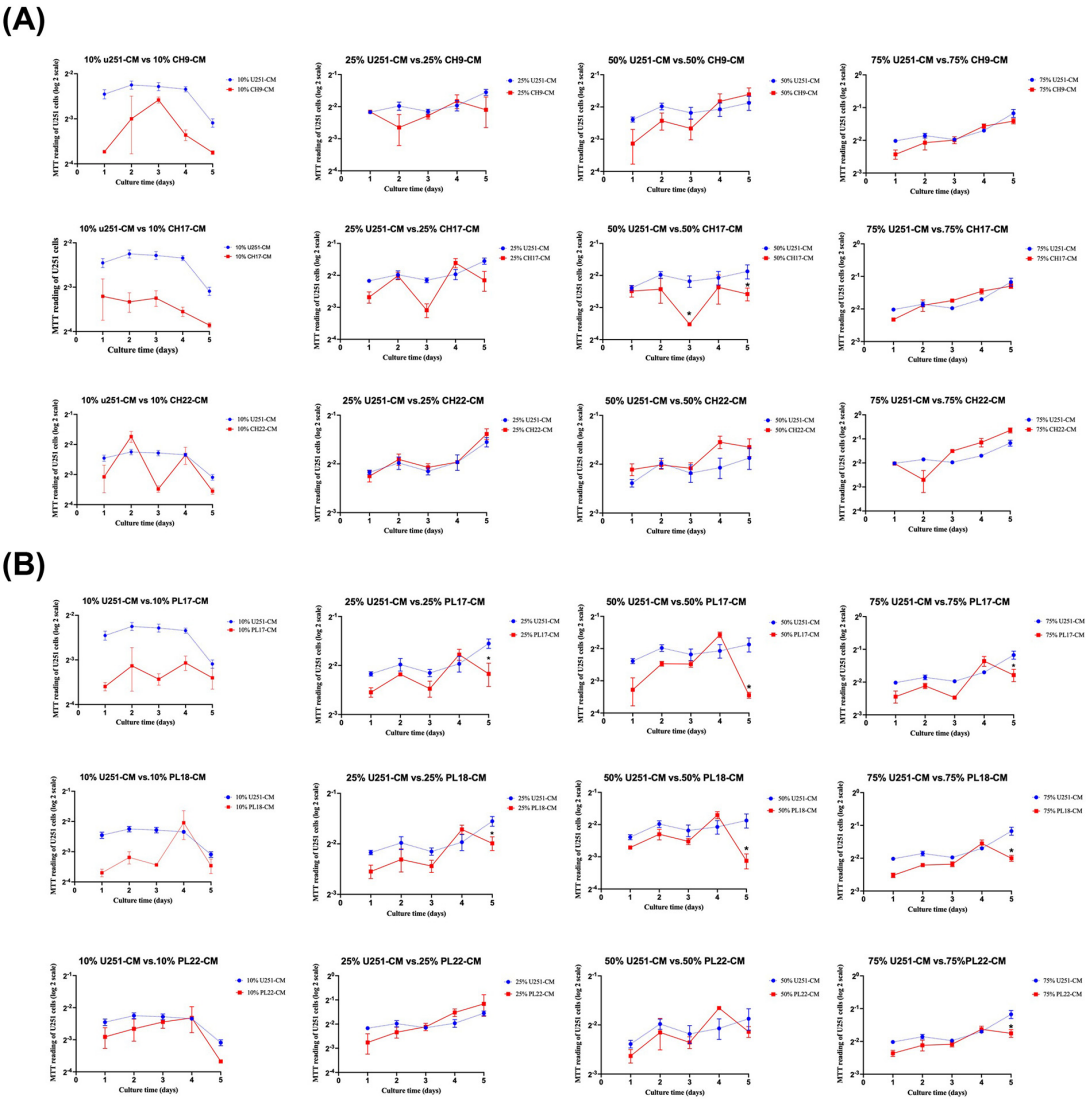
Effect of soluble factors derived from CH-hMSCs and PL-hMSCs on the proliferation of human glioblastoma U251 cells (**A**) Graphs show the growth kinetics of U251 cells cultured in 10–75% (v/v) conditioned media derived from CH-hMSCs (CH-CMs) determined by the MTT assay. (**B**) Graphs show the growth kinetics of U251 cells cultured in 10–75% (v/v) conditioned media derived from PL-hMSCs (PL-CMs) determined by the MTT assay. Data are presented as mean ± SEM of four experiments. U251 cells cultured in DMEM medium + 10% (v/v) FBS served as controls. CH-CMs were prepared from 3 CH-hMSCs (CH9, CH17, and CH22) and PL-CMs were prepared from 3 PL-hMSCs (PL17, PL18, and PL22). *P<0.05 vs control.

The human GBM cell line, U251 cells, were cultured for five days in the various concentrations of conditioned medium made from PL-hMSCs and CH-hMSCs to examine the impact of soluble factors produced from these hMSCs on GBM cell proliferation. The condition media from two out of three cases of CH-hMSCs (CH9-CM and CH22-CM) did not affect U251 cell proliferation. Only 50% CH17-CM inhibited U251 cell proliferation (Figure 1A). Unlike CH-CM, the condition media derived from all three cases of PL-hMSCs (PL-CM) significantly decreased U251 cell proliferation in a dose- and time-dependent manner (Figure 1B).

### The effect of soluble factors derived from hMSCs on U251 cell migration

**Figure 2:**
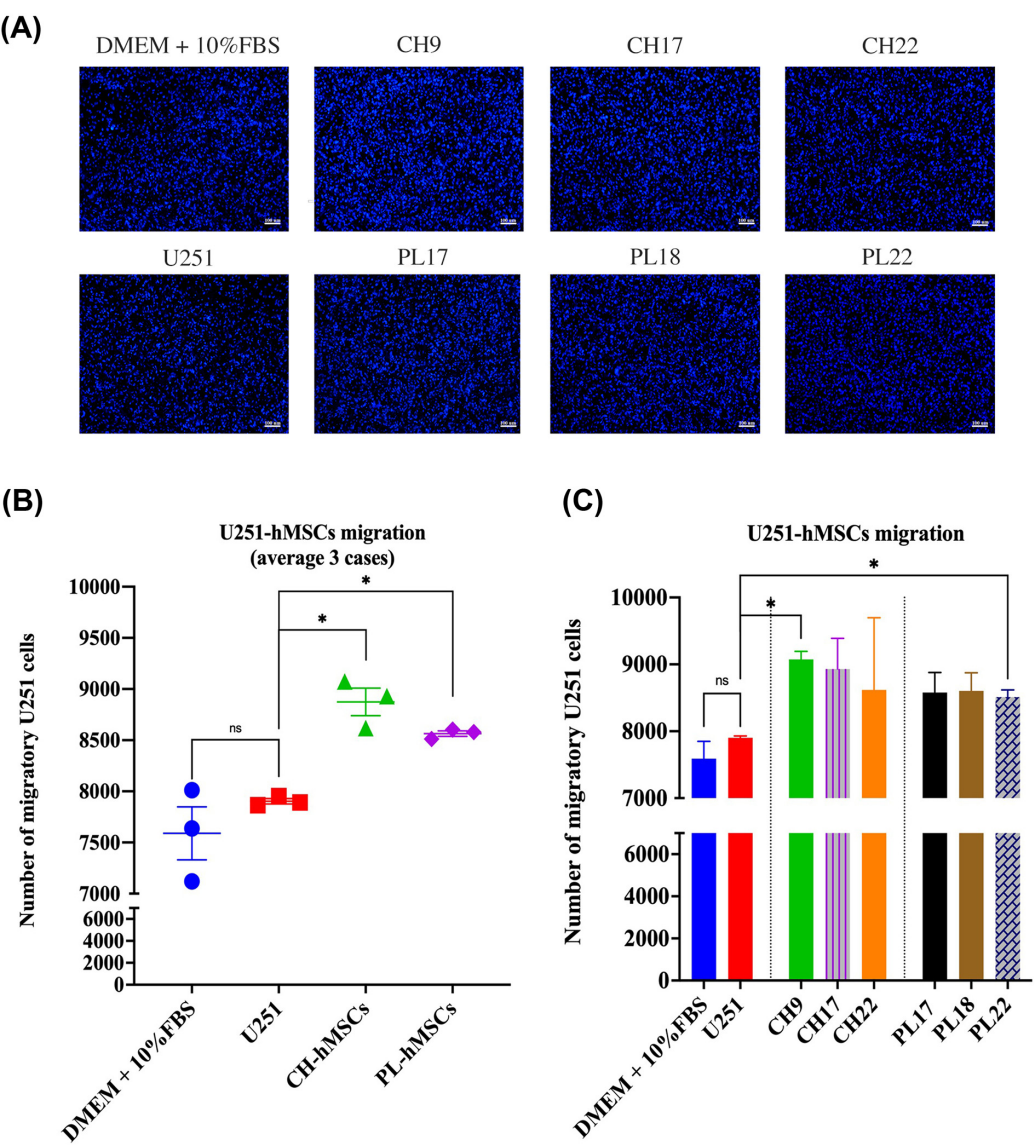
Effect of the soluble factors derived from CH-hMSCs and PL-hMSCs on migration of human glioblastoma U251 cells (**A**) Representative micrographs of U251 cell migration induced by CH-hMSCs and PL-hMSCs determined by the transwell migration assay. CH-hMSCs were derived from three different donors (CH9, CH17, and CH22), and PL-hMSCs were derived from three different donors (PL17, PL18, and PL22). U251 cells induced by U251 cells served as controls (scale bar: 100 µm). (**B**) The graphs show the number of U251 cells that migrate through the transwell after being induced by CH-hMSCs (average of CH9, CH17, and CH22) and PL-hMSCs (average of PL17, PL18, and PL22). Data are presented as mean ± SEM of three hMSCs. U251 induced by U251 cells served as controls; *P<0.05. (**C**) The graphs show the number of migratory U251 cells induced by an individual case of CH-hMSCs and PL-hMSCs. Data are presented as mean ± SEM of three experiments. CH-hMSCs were derived from three different donors (CH9, CH17, and CH22), and PL-hMSCs were derived from three different donors (PL17, PL18, and PL22). U251 cells induced by U251 cells served as controls; *P<0.05.

U251 cells were induced to migrate through a transwell with 5 × 10^5^ PL-hMSCs or CH-hMSCs to assess the impact of soluble factors derived from these two sources of hMSCs on GBM cell migration. The soluble factors released from CH-hMSCs significantly increased U251 cell migration compared with controls induced by 5 × 10^5^ U251 cells (8,874 ± 134 cells vs 7,903 ± 27 cells, *P*<0.05) (Figure 2A,B). Similar to CH-hMSCs, PL-hMSCs also significantly increase U251 cell migration compared with controls (8,564 ± 27 cells vs 7,903 ± 27 cells, *P*<0.05) (Figure 2A,B). These findings imply that the soluble factors produced from these two sources of hMSC improved the ability of U251 cells to migrate. PL-hMSCs and CH-hMSCs derived from three different donors (PL17, PL18, PL22 for PL-hMSCs and CH9, CH17, CH22 for CH-hMSCs) have similar levels of enhancing effect on U251 migration (Figure 2C).

### The effect of hMSC-derived soluble on U251 cell invasion

**Figure 3:**
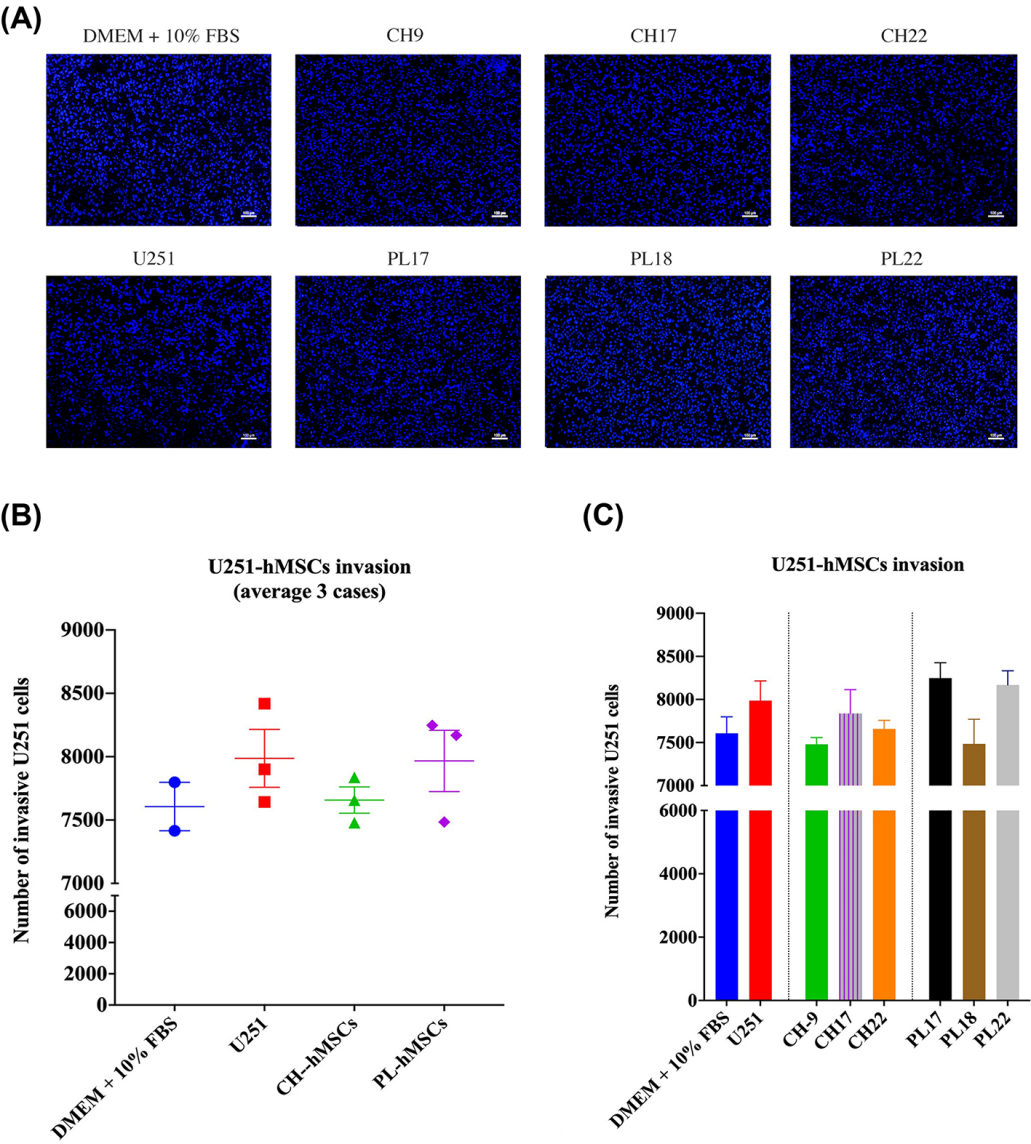
The effect of soluble factors derived from CH-hMSCs and PL-hMSCs on the invasion of human glioblastoma U251 cells (**A**) Representative micrographs of U251 cell invasion induced by CH-hMSCs and PL-hMSCs determined by the transwell invasion assay. CH-hMSCs were derived from three different donors (CH9, CH17, and CH22), and PL-hMSCs were derived from three different donors (PL17, PL18, and PL22). U251 cells induced by U251 cells served as controls (scale bar: 100 µm). (**B**) The graphs show the number of invasive U251 cells after being induced by co-culture with CH-hMSCs (average from CH9, CH17, and CH22) and PL-hMSCs (average from PL17, PL18, and PL22). Data are presented as mean ± SEM of three hMSCs. U251 cells induced by U251 cells served as a control. (**C**) The graphs show the number of invasive U251 cells after co-cultured with an individual case of CH-hMSCs and PL-hMSCs. Data are presented as mean ± SEM of three experiments. CH-hMSCs were derived from three donors (CH9, CH17, and CH22), and PL-hMSCs were derived from three donors (PL17, PL18, and PL22). U251 cells induced by U251 cells served as controls.

Using Matrigel-coated transwells, U251 cells were co-cultured with CH-hMSCs and PL-hMSCs to examine the impact of soluble factors produced from these hMSCs on GBM cell invasion. Differently from their effect on U251 cell migration, CH-hMSCs and PL-hMSCs did not alter U251 cell invasion compared with controls (7,658 ± 103 cells vs 7,987 ± 228 cells and 7,966 ± 242 cells vs 7,987 ± 228 cells, respectively) (Figure 3A,B). PL-hMSCs and CH-hMSCs derived from 3 different donors (PL17, PL18, PL22 for PL-hMSCs and CH9, CH17, CH22 for CH-hMSCs) have a similar effect on the invasion of U251 cells (Figure 3C). These findings imply that although the soluble factors released from these two sources of hMSCs promoted U251 cell migration, their effect on U251 cell invasion is negligible.

### The effect of hMSC-derived soluble factors on U251 cell survival

**Figure 4:**
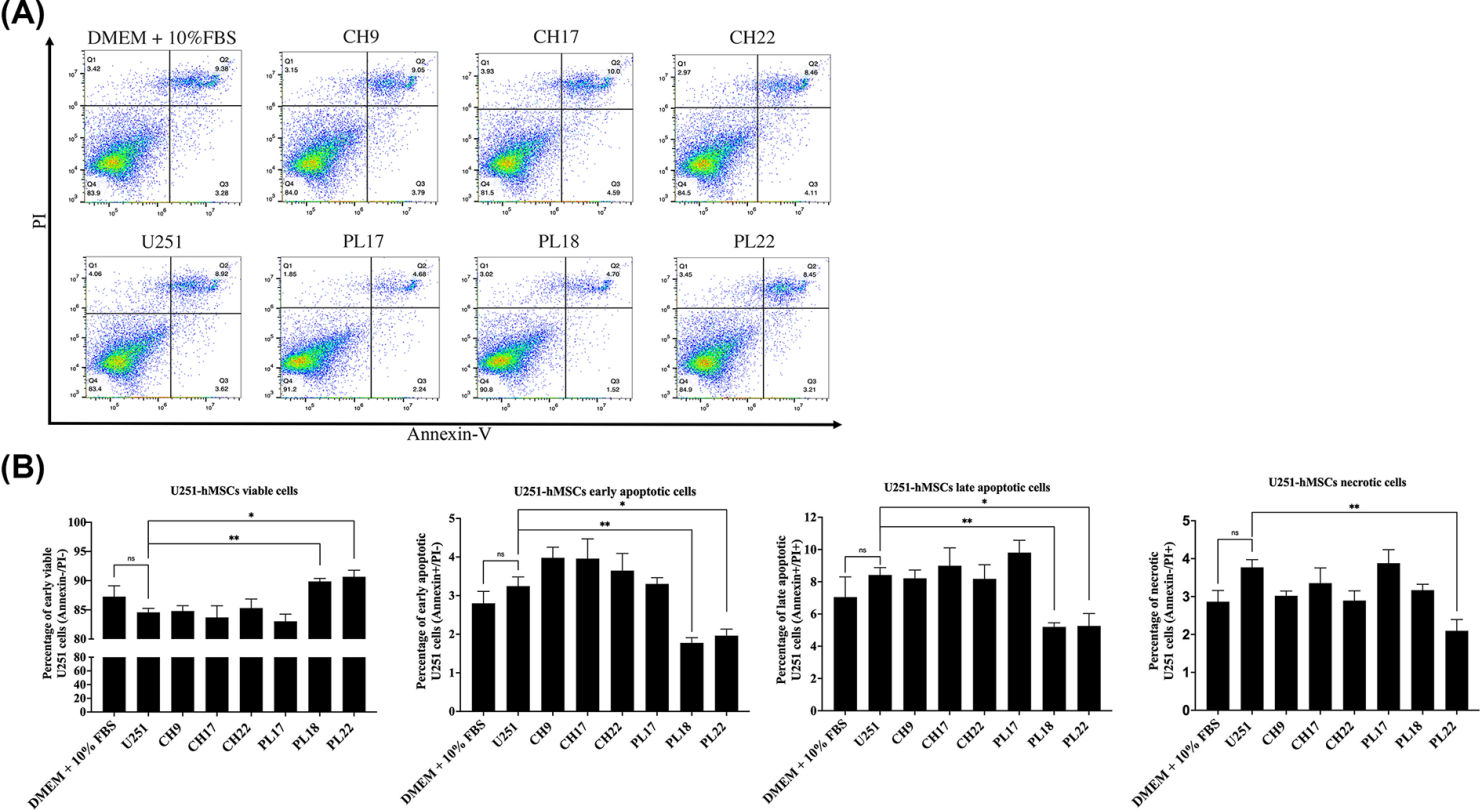
The effect of hMSC-derived soluble factors on U251 cell survival (**A**) Representative flow cytometric dot plot images showing the distribution of U251 cells stained with annexin V and PI after co-culture with various CH-hMSCs and PL-hMSCs. Cells that were positive for annexin V and/or PI staining were considered dead cells. (**B**) Graphs show the percentage of viable, early apoptotic, late apoptotic, and necrotic U251 cells after co-culture with CH-hMSCs and PL-hMSCs as determined by the Annexin V/PI assay. CH-hMSCs and PL-hMSCs were each derived from three donors (CH9, CH17, CH22 and PL17, PL18, PL22). U251 cells co-cultured with U251 cells served as controls. Data are presented as mean ± SEM of three experiments; *P<0.05, **P<0.01.

The effect of hMSC-derived soluble factors on GBM cell survival is determined by co-culture U251 cells with CH-hMSCs or PL-hMSCs using a transwell system for 72 h. The percentages of viable and apoptotic U251 cells were determined using the Annexin V/PI apoptotic assay. The soluble factor derived from CH-hMSCs did not significantly affect the percentages of viable and apoptotic U251 cells compared with the control (Figure 4A,B). Unlike CH-hMSCs, most PL-hMSCs (PL18 and PL22) significantly increased the percentages of viable (89.9 ± 0.5% vs 84.6 ± 0.7%, *P*<0.01 and 90.7 ± 1.1% vs 84.6 ± 0.7%, *P*<0.05, respectively) and reduced the percentages of early apoptotic (1.8 ± 0.1% vs 3.2 ± 0.2%, *P*<0.01 and 2.0 ± 0.2% vs 3.2 ± 0.2%, *P*< 0.05, respectively) and late apoptotic U251 cells (5.2 ± 0.3% vs 8.4 ± 0.5%, *P*<0.01 and 5.3 ± 0.8% vs 8.4 ± 0.5%, *P*<0.05, respectively) compared with control (Figure 4B). Moreover, the soluble factor derived from PL22 also reduced the percentages of necrotic U251 cells compared with the control (2.1 ± 0.3% vs 3.8 ± 0.2%, *P*<0.01) (Figure 4B).

### The effect of hMSC-derived soluble factors on the gene expression of U251 cells

**Figure 5:**
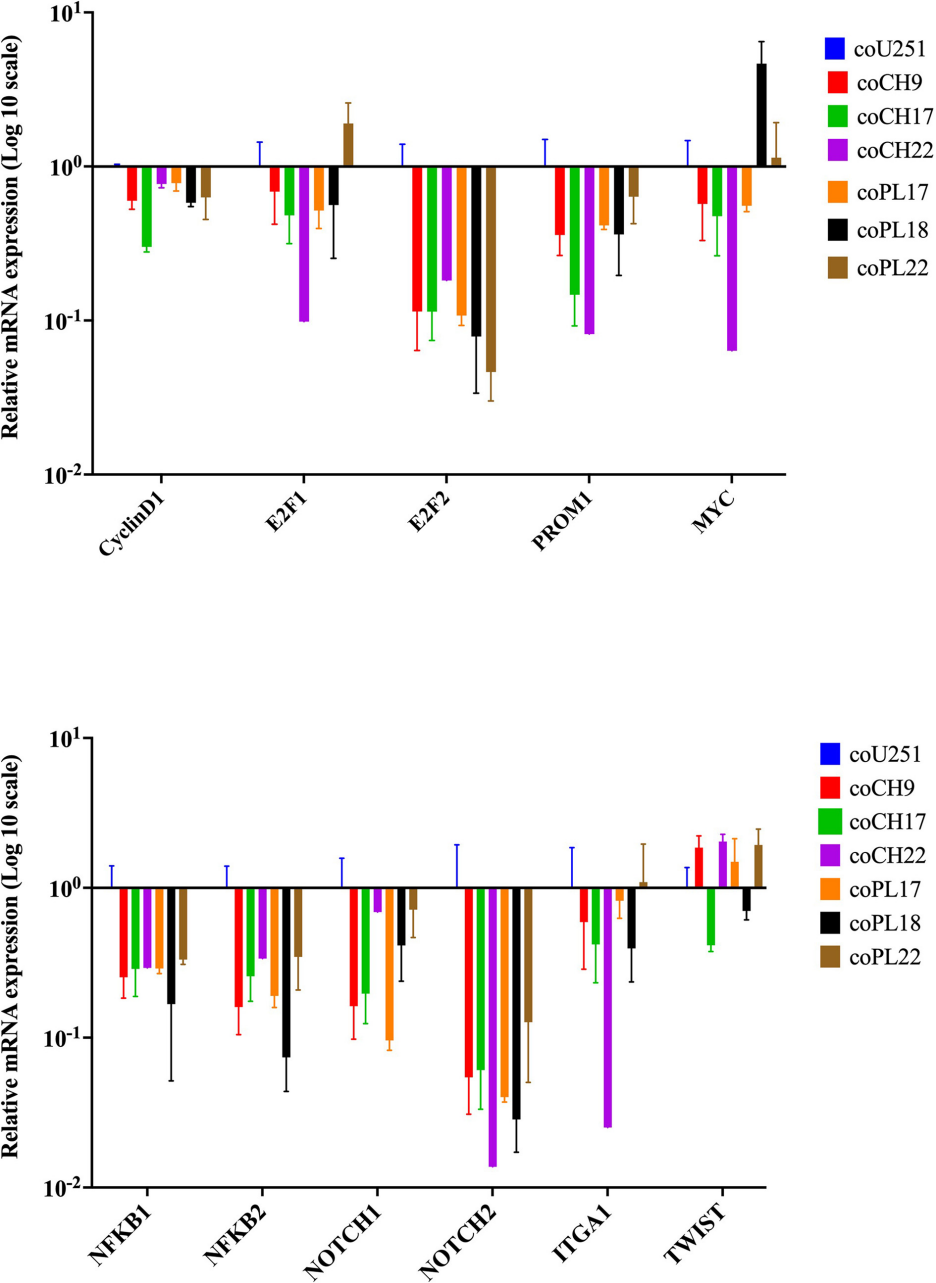
The effect of hMSC-derived soluble factors on the gene expression of human glioblastoma U251 cells The expression levels of genes in U251 cells treated with 75% (v/v) conditioned media derived from CH-hMSCs (coCH) and 75% (v/v) conditioned media derived from PL-hMSCs (coPL) determined by qRT-PCR. CH-hMSC-conditioned media were derived from three CH-hMSCs (CH9, CH17, and CH22), and PL-hMSC-conditioned media were derived from three PL-hMSCs (PL17, PL18, and PL22). U251 cells treated with 75% (v/v) conditioned media derived from U251 cells (CoU251) served as controls. The relative expression levels of the genes in each sample were standardized with the expression level of the same genes in the control samples. Data are presented as mean ± SEM of three experiments.

U251 cells were cultured in 75% (v/v) CH-CMs or 75% (v/v) PL-CMs for 72 h to examine the impact of soluble factors released from these two sources of hMSCs on the expression levels of genes involved in the proliferation and migration of GBM cells. After co-culture, the expression levels of the *CyclinD1*, *E2F1, E2F2, NFKB1, NFKB2, NOTCH1, NOTCH2, PROM1, MYC, TWIST*, and *ITGA1* genes in U251 cells were determined by qRT-PCR. The soluble factors derived from CH-hMSCs and PL-hMSCs decreased the expression levels of several pro-tumorigenic genes, including cell cycle activators (*CyclinD1*, *E2F1, E2F2*, and *MYC*), components of NF-kB signaling pathway (*NFKB1, NFKB2*) and components of Notch signaling pathway (*NOTCH1, NOTCH2*) that are highly activated in GBM cells [27,28], the GBM stem cell marker *PROM1* [29,30], and *ITGA1* that play a role in GBM drug resistant [31] (Figure 5). In contrast, soluble factors from both sources of hMSCs increased the expression level of *TWIST* gene, which plays a crucial role in the epithelial-to-mesenchymal transition and promotes GBM cell migration [32,33] (Figure 5). PL-CMs and CH-CMs from three different donors (PL17, PL18, PL22 for PL-CMs and CH9, CH17, CH22 for CH-CMs) have a similar effect on the gene expression profiles of U251 cells (Figure 5).

### The roles of TGF-β, Wnt/β-catenin, and NF-kB signaling pathways in the effects of hMSC-derived soluble factors

**Figure 6:**
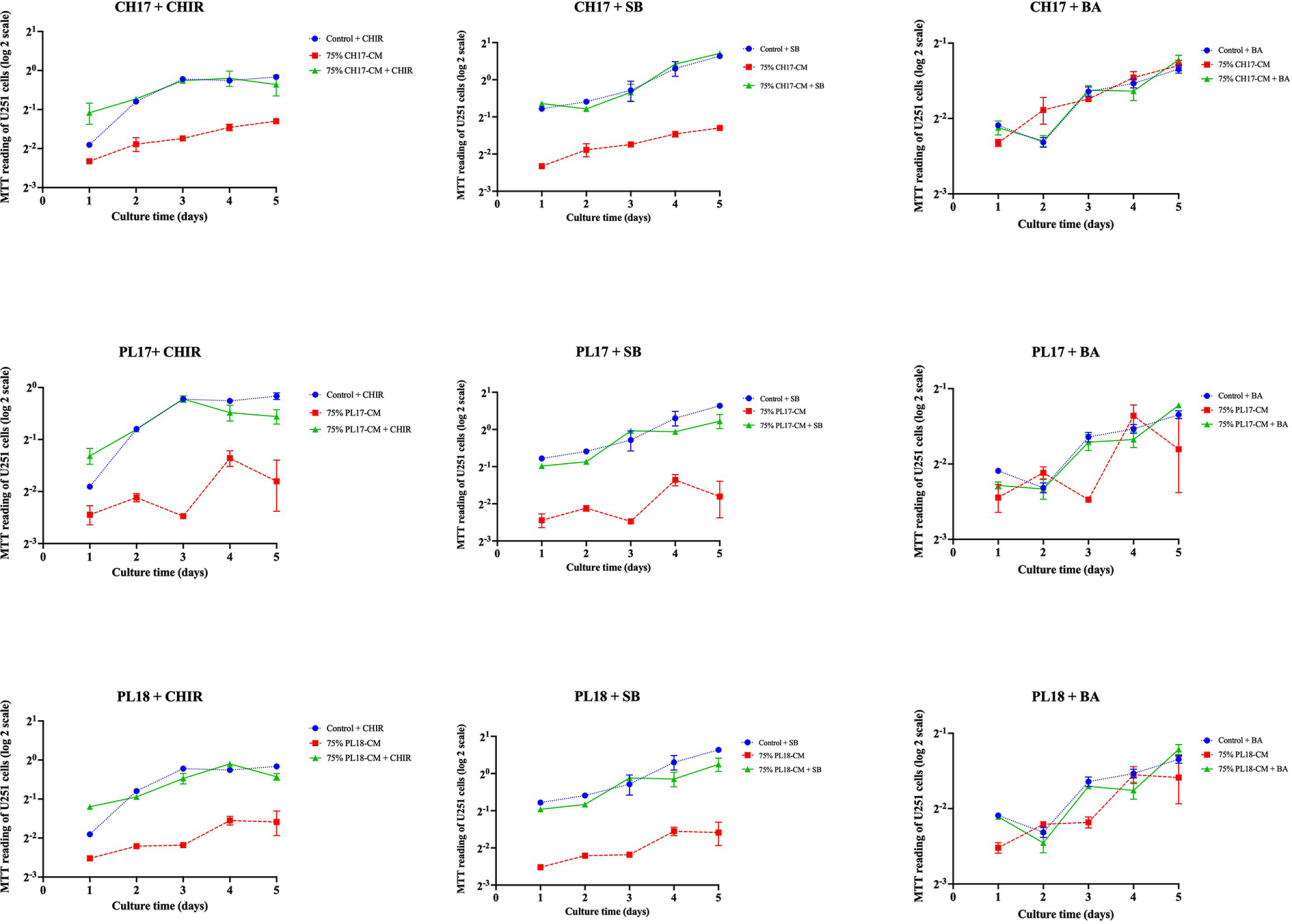
The roles of Wnt/β-catenin, TGF-β, and NF-kB signaling pathways on the proliferation of human glioblastoma U251 cells treated with soluble factors derived from CH-hMSCs and PL-hMSCs The graphs show the growth kinetics of U251 cells cultured in 75% (v/v) conditioned medium derived from CH-hMSCs and PL-hMSCs supplemented with the Wnt activator ChiR99021 (CH-CM + CHIR and PL-CM + CHIR, respectively), TGF-β inhibitor SB431542 (CH-CM + SB and PL-CM + SB, respectively), and the NF-kB activator betulinic acid (CH-CM + BA and PL-CM + BA, respectively), as determined by the MTT assay. Data are presented as mean ± SEM of four experiments. U251 cells cultured in 75% (v/v) conditioned medium derived from CH-hMSCs and PL-hMSCs without CHIR, SB, and BA supplementation served as controls. CH-CM were prepared from CH-hMSCs from one donor (CH17), and PL-CMs were prepared from PL-hMSCs from two donors (PL17 and PL18).

Based on the previous literature [34–47], we hypothesized that TGF-β, Wnt/β-catenin and NF-kB signaling pathways might play a role in the effects of hMSC-derived soluble factors on U251 cells. To investigate this hypothesis, U251 cells were cultured in 75% (v/v) hMSC-CMs supplemented with 3 μM ChiR99021 (CHIR, a Wnt activator), 5 μM SB431542 (SB, a TGF-β inhibitor), or 7 µM betulinic acid (BA, an NF-kB activator) for 5 days. Approximately 75% (v/v) hMSC-CMs were selected for this experiment because they have the highest suppressive effect on U251 cell proliferation (Figure 1). The results show that CHIR and SB significantly decrease the suppressive capacity of hMSC-derived soluble factors on U251 cell proliferation, while BA did not affect this property (Figure 6). These results imply that the inhibitory effect of hMSC-derived soluble factors on U251 cell proliferation may be mediated by suppression of Wnt/β-catenin and activation of TGF-β signaling.

**Figure 7:**
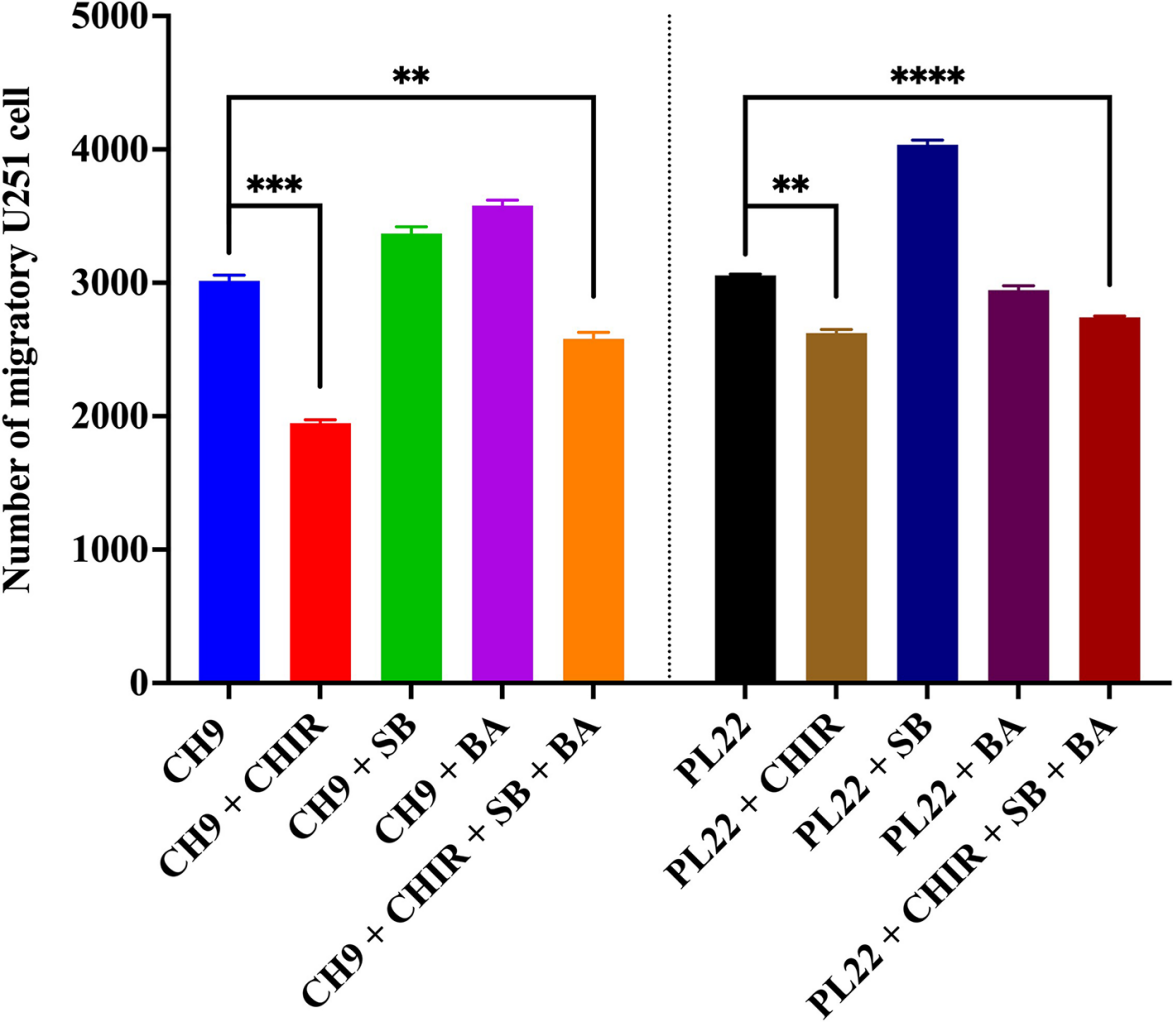
The roles of Wnt/β-catenin, TGF-β, and NF-kB signaling pathways on the migration of U251 cells treated with soluble factors derived from CH-hMSCs and PL-hMSCs Graphs show the number of migratory U251 cells induced by CH-hMSCs and PL-hMSCs in the presence of ChiR99021 (CH9 + CHIR and PL22 + CHIR, respectively), SB431542 (CH9 + SB and PL22 + SB, respectively), and betulinic acid (CH9 + BA and PL22 + BA, respectively), determined by the transwell migration assay. Data are presented as mean ± SEM of three experiments. U251 cells co**-**cultured with hMSCs without CHIR, SB, and BA supplementation (CH9 and PL22) served as controls. In this assay, CH-hMSCs from one donor, CH9, and PL-hMSCs from one donor, PL22, were used. Data are presented as mean ± SEM of three experiments; **P<0.01, ***P<0.001, ****P<0.0001.

Next, we investigate the roles of Wnt/β-catenin, TGF-β, and NF-kB signaling pathways in the migration-inducing effect of soluble factors released from both hMSC sources. U251 cells were co-cultured with CH9 and PL22, which significantly increased U251 cell migration (Figure 2B), using a transwell system in the presence of CHIR, SB and BA for 24 h. The results show that CHIR significantly reduces the ability of CH9 and PL22 to induce U251 migration compared with the controls (1,949 ± 25 cells vs 3,014 ± 43 cells, *P*<0.001 and 2,261 ± 197 cells vs 3,232 ± 197 cells, *P*<0.01, respectively), while SB and BA did not diminish this property (Figure 7). A combination of all three small molecules in both CH9 and PL22 demonstrated effects similar to those of CHIR alone (2,581 ± 48 cells vs 1,949 ± 42 cells and 2,445 ± 220 cells vs 2,261 ± 197 cells, respectively) (Figure 7). These findings suggest that inhibition of Wnt/β-catenin signaling could be responsible, at least in part, for the migration-inducing effect of CH-hMSCs and PL-hMSCs on U251 cells.

**Figure 8:**
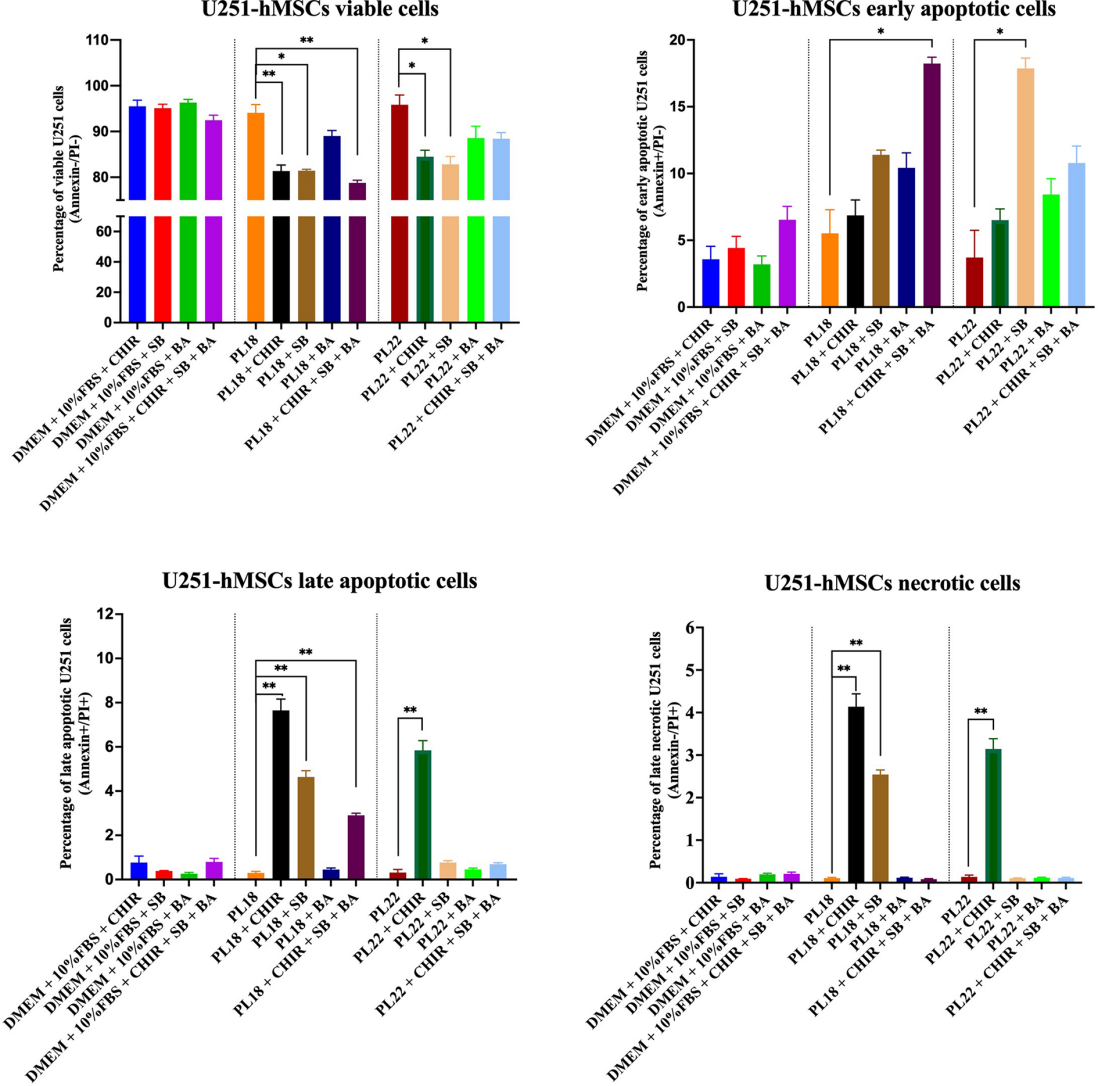
The roles of Wnt/β-catenin, TGF-β, and NF-kB signaling pathways on the survival of U251 cells treated with soluble factors derived from PL-hMSCs Graphs show the percentages of viable, early apoptotic, late apoptotic, and necrotic U251 cells co-cultured with PL-hMSCs in the presence of ChiR99021 (PL18 + CHIR and PL22 + CHIR), SB431542 (PL18 + SB and PL22 + SB), and betulinic acid (PL18 + BA and PL22 + BA), determined by Annexin V/PI assay. Data are presented as mean ± SEM of three experiments. U251 cells co-cultured with PL-hMSCs without CHIR, SB and BA supplementation (PL18 and PL22) served as controls. In this assay, PL-hMSCs from two donors, PL18 and PL22, were used. Data are presented as mean ± SEM of three experiments; *P<0.05, **P<0.01.

To investigate the roles of Wnt/β-catenin, TGF-β, and NF-kB signaling pathways in the survival enhancing effect of soluble factors released from PL-hMSCs, U251 cells were co-cultured with PL18 and PL22, which slightly increased U251 survival (Figure 4B), in the presence of CHIR, SB and BA for 72 h. The Annexin V/PI assay showed that CHIR significantly reduced the percentages of viable U251 cells (81.3 ± 1.3% vs 94 ± 1.8%, *P*<0.01 and 82.8 ± 1.7% vs 95.8 ± 2.1%, *P*<0.05, respectively) and increased the percentages of late apoptotic U251 cells co-cultured with PL18 and PL22 (7.6 ± 0.5% vs 0.3 ± 0.07%, *P*<0.01 and 5.8 ± 0.4% vs 0.3 ± 0.1%, *P*< 0.01, respectively) (Figure 8). Similarly to CHIR, SB significantly reduced the percentages of viable U251 cells co-cultured with PL18 and PL22 (81.4 ± 0.3% vs 94 ± 1.8%, *P*<0.05 and 84.5 ± 1.4% vs 95.8 ± 2.1%, *P*<0.05, respectively) and increased the percentages of late apoptotic U251 cells co-cultured with PL18 (4.6 ± 0.3% vs. 0.3 ± 0.1%, *P*<0.01), while BA did not affect this property (Figure 8). A combination of all three small molecules showed similar effects, but the results were less pronounced than those of CHIR or SB alone (Figure 8). Treatment of U251 cells with CHIR, SB, and BA in DMEM + 10% (v/v) FBS did not affect their survival; therefore, it confirms that the observed effect of CHIR and SB was not caused by their direct toxicity to U251 cells (Figure 8). These findings suggest that inhibition of Wnt/β-catenin signaling and activation of TGF-β signaling might be responsible, at least in parts, for the pro-survival and anti-apoptotic effects of PL-hMSC-derived soluble factors on U251 cells.

## Discussion

The therapeutic potential of hMSCs depends on their ability to produce various factors that affect many physiological and pathological processes, including tissue repair and carcinogenesis [16]. During cancer development, hMSCs have been shown to release several factors, such as insulin-like growth factor 1 (IGF-1), stromal cell-derived factor 1 (SDF-1), TGF-β and vascular endothelial growth factor (VEGF), which play critical roles in promoting tumor growth, improving tumor angiogenesis, and altering tumor immunosurveillance [18–20,48]. Furthermore, a previous study shows that GBM cells could be fused with MSCs resulting in GBM/MSC hybrid cells that exhibit greater proliferative capacity, clonogenicity, and invasive capacity than wild-type MSCs. Furthermore, these hybrid GBM/MSC cells also have greater tumorigenicity than the parental GBM cells and generated larger tumors in nude mice [18]. There is also evidence that GBM cells can transform MSCs by reducing their expression of miR-146a-5p, leading to overexpression of its target gene, heterogeneous nuclear ribonucleoprotein D (*HNRNPD*). These transformed MSCs can subsequently accumulate additional mutations and undergo malignant transformation in the glioma microenvironment [17]. However, a number of other studies indicated that hMSCs inhibited the development and metastasis of various cancers, such as malignant melanoma, colon adenocarcinoma, and hepatocellular carcinoma in mice [26–29]. These conflicting results could be due to the various sources of hMSCs and cancer cells used in these studies, which makes the results of hMSC therapy in cancer patients unreliable and difficult to predict. Therefore, the interaction between GBM cells and MSCs must be fully understood before hMSC transplantation can be used safely to treat these patients.

Our study found that the soluble factors derived from PL-hMSCs and CH-hMSCs have a distinct effect on U251 cell proliferation. While the soluble factors released from PL-hMSCs suppressed proliferation but slightly improved survival of U251 cells, the soluble factors released from most CH-hMSCs, except CH17, did not affect U251 cell proliferation and survival. The hMSCs derived from most tissues, including the chorion and placenta, are a heterogeneous population consisting of several subpopulations that release different combinations of soluble factors. Although PL-hMSCs exhibit a typical hMSC characteristic similar to those of CH-hMSCs, these two hMSC sources were derived from different tissues. Therefore, PL-hMSCs may release a different combination of soluble factors that have a more suppressive effect on U251 cell proliferation compared to those released from CH-hMSCs.

According to the gene expression study, hMSC-derived soluble factors could inhibit U251 cell proliferation by downregulating the expression level of many pro-tumorigenic genes, including *CyclinD1*, *E2F1, E2F2, NFKB1, NFKB2, NOTCH1, NOTCH2, PROM1, MYC*, and *ITGA1* in these cells. It has been demonstrated that each of these genes is essential for the development and progression of GBM [49–52]. The suppressive effect of soluble factors derived from CH-hMSCs and PL-hMSCs on GBM cell proliferation is consistent with previous publications showing that BM-hMSCs, a common source of hMSCs for clinical applications, inhibit proliferation and induce cell cycle arrest in U251 cells [53] and Ad-hMSCs derived from adipose tissue, another common source of hMSCs for clinical applications, inhibit the growth of human GBM xenograft in rats [54]. In addition, other previous studies also showed that soluble factors released from BM-hMSCs, Ad-hMSCs, and Wharton’s jelly derived WJ-hMSCs suppressed the proliferation, inhibited cell-cycle progression and induced apoptosis of U251 and U87 cells, which is another commonly used GBM cell line [55,56]. This evidence suggests that CH-hMSCs and PL-hMSCs release soluble factors that suppress the proliferation of human GBM cells by down-regulating the expression level of many pro-tumorigenic genes and could potentially be used as a substitute for BM-hMSCs and Ad-hMSCs, which requires an invasive method for their harvest, in cancer treatment.

Unlike their effect on GBM cell proliferation, soluble factors derived from both hMSC sources increased U251 cell migration, possibly by up-regulating the expression level of *TWIST* which has been shown to promote epithelial-to-mesenchymal transition (EMT) and migration of many cancer cells, including GBM [57–60]. It should be noted that while both sources of hMSCs increased U251 cell migration, they did not have an impact on U251 cell invasion. These findings provided evidence that the soluble factors that induce U251 cells to migrate and invade may be different. Cell invasion requires a set of proteins that is different from cell migration, especially those involved in the degradation of extracellular matrix proteins, such as matrix metalloproteinases [61], serine proteinase [62], and cathepsins [63] that are not critical to cell migration. Therefore, although the soluble factors derived from CH-hMSCs and PL-hMSCs significantly increased the migration of U251 cells, they may not necessarily affect the invasion of U251 cells. This result contradicts the previous studies showing that BM-hMSCs reduced U251 cell migration and invasion by suppressing PI3K/AKT signaling and inhibiting its EMT [53] and Ad-hMSCs and WJ-hMSCs suppressed U87 cell migration [55]. This discrepancy is probably caused by the difference in the sources of hMSCs used in each study, since other previous studies showed that soluble factors derived from Ad-MSCs enhanced C6 rat glioma cell migration and promoted their EMT [64], and soluble factors derived from glioma-associated hMSCs (gaMSCs) induced EMT and increased U87 cell migration by up-regulating *FOXS1* expression [65]. Because GBM primarily migrates into surrounding brain tissue and usually does not metastasize to other far away regions, it is possible that increasing GBM cell migration might also contribute to its aggressiveness. Nonetheless, these results should be confirmed by *in vivo* tumor migration and invasion assays.

TGF-β and NF-kB signaling pathways have been shown to be essential in the development and progression of GBM by regulating the expression of genes involved in its growth, survival, and invasion [34–41]. Similarly, Wnt/β-catenin signaling is another pathway that has been found to be involved in the development and progression of GBM by improving its growth and migration [42–47]. Based on these previous publications, we hypothesized that Wnt/β-catenin, TGF-β, and NF-kB signaling pathways might play a role in the effects of hMSCs on U251 cell proliferation, migration and survival. The functional study using ChiR99021, SB431542 and betulinic acid suggests that CH-hMSCs and PL-hMSCs suppressed U251 cell proliferation, at least in part, through the activation of TGF-β, and inhibition of Wnt/β-catenin signaling pathways. This result is consistent with a previous study showing that umbilical cord-derived UC-hMSCs suppressed the proliferation of rat C6 glioma cells by releasing DKK1, an inhibitor of Wnt/β-catenin pathway [66]. In addition, we also showed that inhibition of Wnt/β-catenin pathway could also mediate the migration-inducing effect of PL-hMSCs and CH-hMSCs on U251 cells. Furthermore, soluble factors derived from PL-hMSCs also slightly improve U251 cell survival through activation of TGF-β and inhibition of Wnt/β-catenin signaling pathways. Unlike TGF-β and Wnt/β-catenin signaling, our results suggest that NF-kB signaling pathway does not play a significant role in the effects of hMSCs on U251 cell proliferation, migration, and survival.

## Conclusion

Our study shows that CH-hMSCs and PL-hMSCs inhibited GBM cell proliferation by down-regulating the expression of many pro-tumorigenic genes. The suppressive effect of PL-hMSCs on U251 proliferation could be mediated, at least in part, by inhibition of Wnt/β-catenin and activation of TGF-β signaling in these cells. On the contrary, inhibition of Wnt/β-catenin and activation of TGF-β by the soluble factors derived from both sources of hMSCs could enhance the migration of GBM cells, possibly by up-regulating the expression of *TWIST*, a well-known EMT inducer. Our study provides a better understanding of the interaction between GBM cells and gestational tissue-derived hMSCs. This knowledge might be used to develop safer and more effective stem cell therapy that improves the survival and quality of life of patients with GBM by manipulating the interaction between hMSCs and GBM cells.

## Supplementary Material

Supplementary Figure S1

## Data Availability

All supporting data are included within the main article and its supplementary files.

## Abbreviations

BA: betulinic acid
CHIR: ChiR99021
CH-CM: conditioned media derived from CH-hMSCs
CH-hMSC: chorion-derived human mesenchymal stem cell
CoCH: U251 cells co-cultured with 75% conditioned media derived from CH-hMSCs
CoCH: U251 cells treated with 75% conditioned media
CoPL: U251 cells co-cultured with 75% conditioned media derived from PL-hMSCs
CoU251: U251 cells co-cultured with 75% conditioned media derived from U251 cells
PL-CM: conditioned media derived from PL-hMSCs
PL-hMSC: placenta-derived human mesenchymal stem cell
SB: SB431542
