# Supplementary material for: PL-hMSC and CH-hMSC derived soluble factors inhibit proliferation but improve hGBM cell migration by activating TGF-β and inhibiting Wnt signaling
Source: Biosci Rep. 2024 May 17;44(5):BSR20231964. doi: 10.1042/BSR20231964 (PMC11130542; doi:10.1042/BSR20231964)
Supplement: Supplementary Figure S1 [file BSR-2023-1964_supp.pdf]

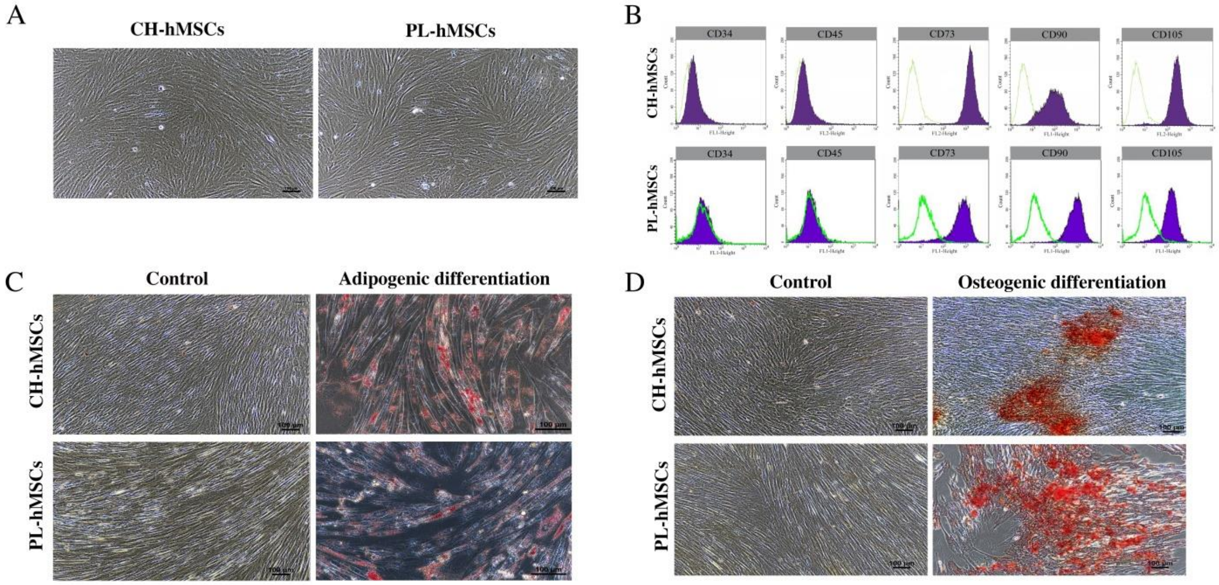


Supplementary Figure 1: Characteristics of CH-hMSCs and PL-hMSCs.

A) Fibroblast-like morphology of CH-hMSCs and PL-hMSCs. (Scale bar: 100 µm)

B) Immunophenotypes of CH-hMSCs and PL-hMSCs as determined by flow cytometry.

C) Adipogenic differentiation of CH-hMSCs and PL-hMSCs as determined by oil-red O staining. (Scale bar: 100 µm)

D) Osteogenic differentiation of CH-hMSCs and PL-hMSCs as determined by alizarin red S staining. (Scale bar: 100 µm)
